# Low-Threshold Anti-Stokes Raman Microlaser on Thin-Film Lithium Niobate Chip

**DOI:** 10.3390/ma17051042

**Published:** 2024-02-24

**Authors:** Jianglin Guan, Jintian Lin, Renhong Gao, Chuntao Li, Guanghui Zhao, Minghui Li, Min Wang, Lingling Qiao, Ya Cheng

**Affiliations:** 1 State Key Laboratory of Precision Spectroscopy, East China Normal University, Shanghai 200241, China; 51200920051@stu.ecnu.edu.cn (J.G.); 52270920001@stu.ecnu.edu.cn (C.L.); 2 The Extreme Optoelectromechanics Laboratory (XXL), School of Physics and Electronic Science, East China Normal University, Shanghai 200241, China; rhgao@siom.ac.cn (R.G.); mwang@phy.ecnu.edu.cn (M.W.); 3 State Key Laboratory of High Field Laser Physics and CAS Center for Excellence in Ultra-Intense Laser Science, Shanghai Institute of Optics and Fine Mechanics (SIOM), Chinese Academy of Sciences (CAS), Shanghai 201800, China; zhaogh2022@shanghaitech.edu.cn (G.Z.); minghuili@siom.ac.cn (M.L.); qiaolingling@siom.ac.cn (L.Q.); 4 Center of Materials Science and Optoelectronics Engineering, University of Chinese Academy of Sciences, Beijing 100049, China; 5 School of Physical Science and Technology, Shanghai Tech University, Shanghai 200031, China; 6 Collaborative Innovation Center of Extreme Optics, Shanxi University, Taiyuan 030006, China; 7 Collaborative Innovation Center of Light Manipulations and Applications, Shandong Normal University, Jinan 250358, China; 8 Shanghai Research Center for Quantum Sciences, Shanghai 201315, China; 9 Hefei National Laboratory, Hefei 230088, China

**Keywords:** stimulated Raman scattering, stimulated anti-Stokes Raman scattering, lithium niobate, whispering gallery modes, optical microcavity

## Abstract

Raman microlasers form on-chip versatile light sources by optical pumping, enabling numerical applications ranging from telecommunications to biological detection. Stimulated Raman scattering (SRS) lasing has been demonstrated in optical microresonators, leveraging high Q factors and small mode volume to generate downconverted photons based on the interaction of light with the Stokes vibrational mode. Unlike redshifted SRS, stimulated anti-Stokes Raman scattering (SARS) further involves the interplay between the pump photon and the SRS photon to generate an upconverted photon, depending on a highly efficient SRS signal as an essential prerequisite. Therefore, achieving SARS in microresonators is challenging due to the low lasing efficiencies of integrated Raman lasers caused by intrinsically low Raman gain. In this work, high-Q whispering gallery microresonators were fabricated by femtosecond laser photolithography assisted chemo-mechanical etching on thin-film lithium niobate (TFLN), which is a strong Raman-gain photonic platform. The high Q factor reached 4.42 × 10^6^, which dramatically increased the circulating light intensity within a small volume. And a strong Stokes vibrational frequency of 264 cm^−1^ of lithium niobate was selectively excited, leading to a highly efficient SRS lasing signal with a conversion efficiency of 40.6%. And the threshold for SRS was only 0.33 mW, which is about half the best record previously reported on a TFLN platform. The combination of high Q factors, a small cavity size of 120 μm, and the excitation of a strong Raman mode allowed the formation of SARS lasing with only a 0.46 mW pump threshold.

## 1. Introduction

Since the discovery of spontaneous Raman scattering by C. V. Raman in 1928, Raman spectroscopy has emerged as a pivotal instrument for investigating the intricate interplay between light and matter [1,2,3,4,5,6,7,8]. And Raman lasers based on the stimulated Raman scattering (SRS) process featuring high conversion efficiency and superior beam quality have undergone rapid advancement to extend the available spectral coverage of the coherent semiconductor light sources and ultrafast fiber lasers from infrared to ultraviolet wavelengths, yielding a wealth of scientific and technological breakthroughs thanks to the invention of the laser by Maiman in 1960. These remarkable characteristics of Raman lasers have propelled their widespread utilization across a multitude of disciplines, including light detection and ranging (LIDAR), atmospheric monitoring, laser scanning, optical frequency conversion, Raman laser spectroscopic analysis, telecommunications, precision metrology, and bio-detection [1,2,3,4,5], which often require high pump powers in macroscale devices. However, these traditional macroscale devices face challenges in terms of size, manufacturing cost, high pump consumptions, and integration scalability. And there is an ever-growing demand to move from benchtop to chip-level platforms, offering the prospect of on-chip laser sources in photonic integration, with low pump power and low manufacturing cost [1,2,3,4,5], by leveraging the strong light-field confinement in photonic structures fabricated by micro/nanofabrication techniques and functional scalability. These advancements have paved the way for the development of compact and efficient coherent light sources on integrated photonic devices, which have showcased their potential in photonic applications such as optical communications, sensing, and spectroscopy [6,7,8]. 

To this end, much endeavor has been made using integrated whispering gallery microresonators with high Q factors, which allow a large built-up light intensity within a small mode volume, leading to low-threshold SRS for providing downconverted laser signals [1,2,3,4,5]. As SRS microlasers originate from a weak third-order nonlinear interaction of light with the Stokes vibrational modes of scattering platforms [9,10,11,12], the SRS lasing efficiency/power is often poor. Similar to SRS, the stimulated anti-Stokes Raman scattering (SARS) process is also crucial for generating upconverted laser signals. However, unlike SRS, SARS heavily depends on the interaction between the pump photons and the SRS signal [9]. As a result, achieving SARS microlasers is more challenging because of the necessary additional requirement of a sufficient population of photons at both the SRS and pump wavelengths.

One way to directly generate SARS microlasers is to employ high-pump-power lasing for increasing the pump photons and the SRS photons, akin to benchtop anti-Stokes Raman lasers [13,14,15,16]. However, this approach will face hurdles to being readily available as chip-level photonic integration in the future [17,18]. Therefore, to generate SARS microlasers, metal dopant has been doped in ultra-high-Q (Q > 10^7^) silica microresonators to increase the Raman gain of the cavity material (i.e., silica) and decrease the mode volume to increase the SRS efficiency [9], at the expense of the significant increase in manufacturing complicacy and difficulty, which cannot be easily extended to other photonic platforms. Compared with silica, thin-film lithium niobate (TFLN) is a much stronger Raman-active medium and is more favored for photonic integrated platforms because of its outstanding properties featuring high second-order nonlinearity, large Pockels electro-optic coefficient, and moderate refractive index [17]. Numerous photonic devices have been demonstrated on the TFLN platform with high performances, ranging from high-speed electro-optical modulators, efficient nonlinear optical frequency convertors, bright quantum light sources, soliton frequency combs, meter-scale length optical waveguide true delay lines, narrow-linewidth microlasers, high-gain waveguide amplifiers, and large-scale photonic integrated circuits [17,19,20,21,22,23,24,25,26,27,28,29,30,31,32,33,34,35,36,37]. Moreover, SRS microlasers [4,37,38,39] have also been operated on the TFLN platform with a threshold as low as 0.62 mW [39], resulting from the fabrication of ultra-high-Q microresonators by femtosecond laser photolithography assisted chemo-mechanical etching (PLACE) [37] to significantly increase the circulating light intensities. However, thanks to the excitation of the relatively weak Raman vibrational frequency of 690 cm^−1^ [40], anti-Stokes Raman microlasers have not yet been demonstrated on the TFLN platform due to the requirement of both the ultra-high Q factors and excitation of the strong Raman vibrational frequency. 

In this work, the generation of SRS and SARS was demonstrated in TFLN microresonators by dispersion engineering for the first time. The dispersion-engineered TFLN microresonators fabricated by the femtosecond laser PLACE technique provided ultra-high Q (~4.42 × 10^6^) modes that are aligned to the pump wavelength and the Raman signal photon involved with the excitation of a strong Raman phonon branch of 264 cm^−1^ of lithium niobate crystal, leading to a significant cavity enhancement in the efficiencies of SRS and SARS. The SRS efficiency reached 40.6% with a reduced threshold of only 0.33 mW, presenting the state of the art in the TFLN platform [4,37,38,39]. Furthermore, the SARS efficiency was measured to be 1.7% with a low threshold of 0.46 mW, attributed to the ultra-high-Q microresonator and the increased Raman gain by selectively exciting the strong phonon branch. This work provides a route to the generation of low-threshold, coherent upconverted light sources for lots of wavelength bands that are difficult to access on-chip.

## 2. The Fabrication of the High-Q TFLN Microdisk by the PLACE Technique

**Figure 1:**
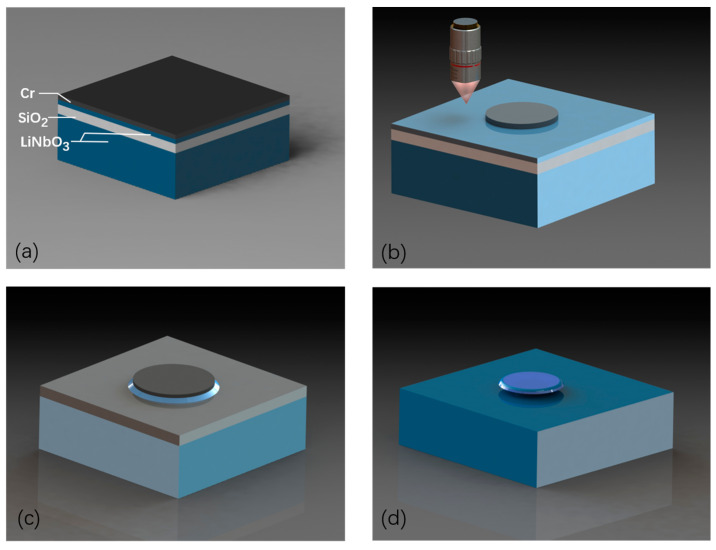
A schematic illustration of the processing flow for the fabrication of the TFLN microdisk as follows: (**a**) A layer of chromium (Cr) is uniformly deposited onto the TFLN wafer (LiNbO_3_ layer). (**b**) The femtosecond pulsed laser ablates the Cr layer into the desired microdisk pattern. (**c**) The Cr pattern is transferred to the TFLN through a chemo-mechanical polishing process, resulting in smooth and well-defined microdisk structures. (**d**) The Cr etching solution is employed to remove the Cr mask, and the silicon dioxide (SiO_2_) underneath the microdisk is selectively undercut in buffered HF solution, leading to the formation of suspended TFLN microdisks with the desired geometry.

The dispersion-engineered TFLN microdisks were fabricated on commercially available X-cut TFLN wafer with a thickness of 700 nm (NANOLN, Jinan Jingzheng Electronics Co., Ltd., Jinan, China). This TFLN thin film was bonded to a 2 μm thick silicon dioxide layer on a 500 μm thick lithium niobate handle wafer. The diameter of the microdisk was designed to be approximately 120 μm. Then the microdisks were fabricated by the femtosecond laser PLACE technique with 5 continuous steps [37], as illustrated in Figure 1. First, a 600-nm thick chromium (Cr) layer was deposited onto the TFLN wafer using magnetron sputtering. Second, an ultrafast femtosecond pulsed laser with a center wavelength of 1030 nm and pulse width of ~190 fs (PHAROS, LIGHT CONVERSION Inc., Vilnius, Lithuania) was employed to only subtractively ablate the Cr layer into Cr disk-shape patterns with 200 nm spatial resolution. The TFLN remained intact during the laser ablation, as its ablation threshold is much higher than that of Cr metal. This Cr pattern served as a hard mask to protect the underneath TFLN material in the next step. Subsequently, the sample underwent chemo-mechanical polishing to etch the exposed TFLN material using a wafer polishing machine (UNIPOL802, Kejing Inc., Shenyang, China), resulting in the formation of TFLN microdisks via pattern transferring from the Cr layer to the TFLN layer. Then, the residual Cr mask above the TFLN microdisks was removed using a Cr etching solution (Chromium etchant, Alfa Aesar GmbH, Ward Hill, MA, USA). Finally, the sample endured secondary chemo-mechanical polishing to further improve the smooth surface, and the silicon dioxide layer underneath the TFLN microdisks was undercut into small pillars to support the suspended TFLN microdisks with wet etching in buffered HF solution by carefully controlling the time of etching.

**Figure 2:**
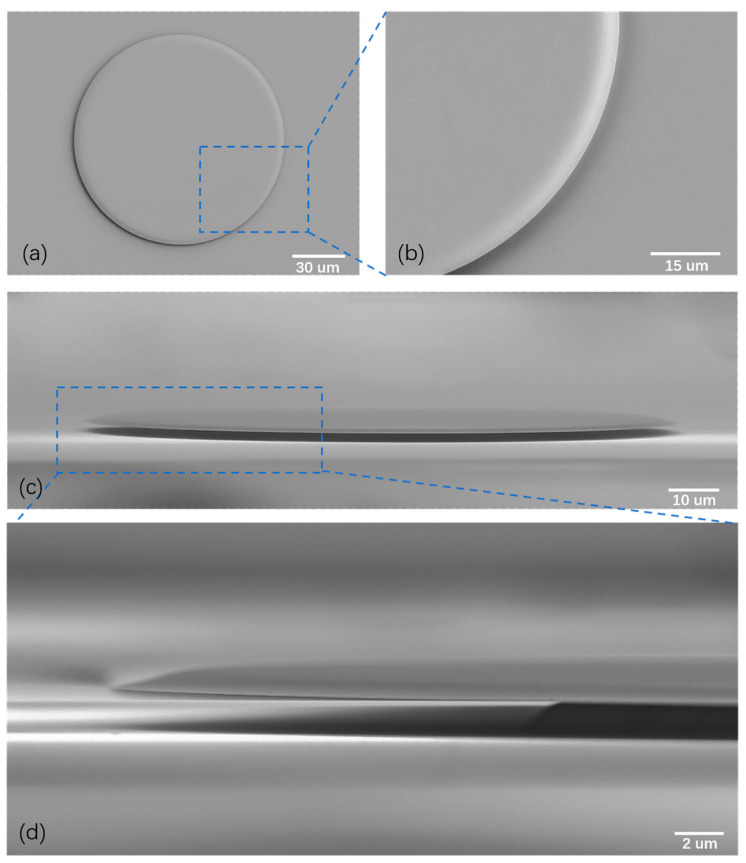
SEM images of the fabricated TFLN microdisk. (**a**) Top-view image, showing its entire structure. (**b**) Enlarged view image of the blue dashed box in (**a**), highlighting the smooth sidewall. (**c**) Side-view SEM image, exhibiting the cross-section of the microdisk. (**d**) Enlarged view of the blue dashed box in (**c**), exhibiting that the periphery of the microdisk is far from the rough pillar boundary with a distance of 18 μm.

Figure 2 plots the scanning electron microscope (SEM) images of the fabricated suspended microdisk. The microdisk possesses a diameter of approximately 120 μm, a thickness of ~700 nm, and a wedge angle of the side wall of ~19°. In Figure 2a, the top-view SEM image of the microdisk reveals a remarkably smooth surface. Notably, the microdisk edge also exhibits a smooth sidewall, as shown in Figure 2b. This smoothness of the total internal reflection interface and the flat gradient of the side wall with a typical wedge angle of ~19% play pivotal roles in maintaining an ultra-high Q factor of the fabricated microdisk, which is crucial for the observation of efficient SRS and SARS within the microdisk. Moreover, the side-view SEM image of the microdisk shows that the periphery of the microdisk is far from the rough boundary of the silicon dioxide pillar with a distance of ~18 μm, as depicted in Figure 2c,d. This large distance is desired to mitigate the reduction in the Q factor of the whispering gallery modes (WGMs) within the microdisk caused by scattering loss from the rough pillar boundary. The combination of the ultrasmooth total internal reflection interface and the large distance between the periphery of the microdisk and the pillar boundary ensures ultra-high-Q WGMs within the microdisk.

## 3. The Experimental Setup for Low-Threshold SRS and SARS in the Microdisk

**Figure 3:**
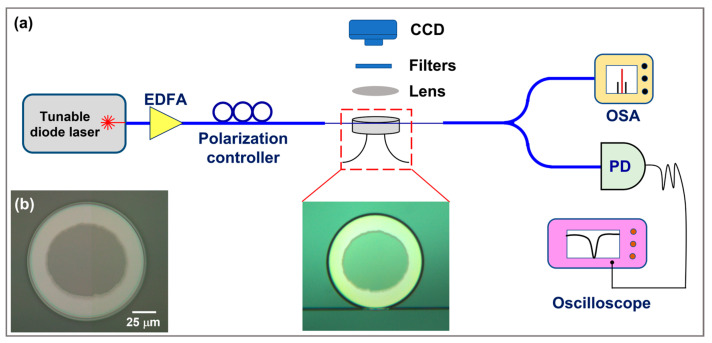
(**a**) Experimental setup for the generation of SRS and SARS lasing. Inset: the optical micrograph of the microdisk coupled with the tapered fiber. Here, optical spectral analyzer is denoted as OSA. (**b**) Optical microscope image of the TFLN suspended microdisk.

The experimental setup for low-threshold SRS and SARS in the microdisk is schematically illustrated in Figure 3. A continuous-wave laser diode (Model: TLB-6728, New Focus, Inc., San Jose, CA, USA) with a narrow-linewidth provided a pump source with tunable wavelength ranging from 1530 to 1570 nm, and the output power was subsequently amplified by an erbium-doped fiber amplifier (EDFA). The long-term linewidth of the laser diode was narrower than 200 kHz. An inline polarization controller was inserted to finely adjust the polarization state of the pump light. Then the pump light was sent into a tapered fiber with a waist of 2 μm, which was used to over-couple with the fabricated microdisk to excite the WGMs. Here, the coupling position between the tapered fiber and the microdisk was precisely controlled by a three-dimensional piezoelectric stage with 5 nm resolution. To facilitate real-time monitoring of the coupled position and capture the light emission from the microdisk, an optical microscope imaging system, which consists of a charge-coupled device (CCD) camera and an objective lens with a numerical aperture of 0.28, was built above the microdisk. The generated nonlinear signals were coupled out of the microdisk via the same tapered fiber. The output signals from the tapered fiber were separated by a 1 × 2 single-mode fiber splitter with a power ratio of 20/80. The 20% light beam was sent into an optical spectral analyzer (OSA) to analyze the optical spectrum. The minimum spectral resolution of the OSA (Model: AQ6370D, Yokogawa Inc., Tokyo, Japan) was 0.02 nm, the detection spectral window of the OSA ranged from 600 to 1700 nm, and the detection sensitivity was down to −70 dBm. At the same time, the other 80% light beam was sent into a fast photodetector (PD) to convert the optical signal into an electrical signal for recording the transmission spectrum using an oscilloscope during mode characterization. The microdisk coupled with the tapered fiber is shown in the inset of Figure 3, which shows the tapered fiber was placed in contact with the microdisk side wall to achieve a stable coupling and efficiently excite the WGMs within the microdisk. All the participating waves were controlled to be transverse-electrically polarized. To measure the loaded Q factors of WGMs, the pump power was reduced to ~5 μW to avoid triggering thermal-optic and photo-refractive effects, and a triangular wave signal was used to drive the pump laser to scan the laser wavelength around the resonant wavelength, leading to a resonant dip signal in the oscilloscope. Figure 3b shows the optical micrograph of the microdisk.

## 4. The Generation of Low-Threshold SRS and SARS Microlasers

**Figure 4:**
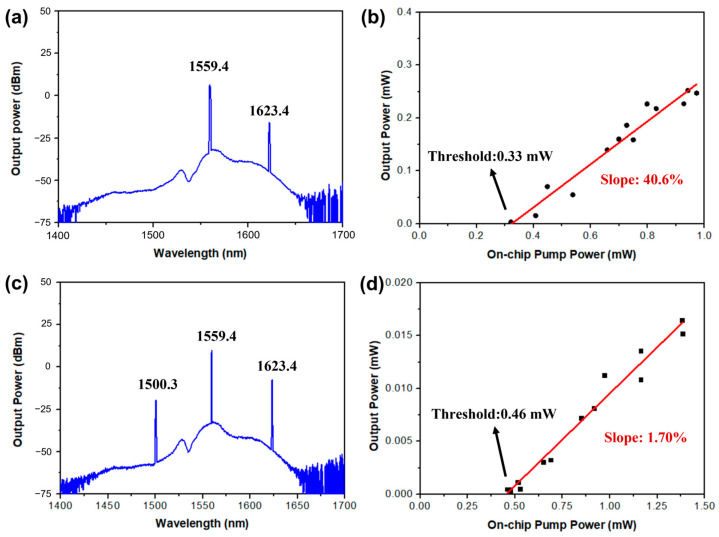
(**a**) The spectrum of the generated SRS signal at 1623.4 nm wavelength. (**b**) The dependence of the Stokes Raman laser peak power on the on-chip pump power (black dots), exhibiting a threshold of only 0.33 mW and a linear growth slope of 40.6% above the threshold by linearly fitting (red line). (**c**) The spectrum of the generated SRS and SARS signals at wavelengths of 1623.4 and 1500.3 nm, respectively. (**d**) The dependence of the anti-Stokes Raman laser peak power on the on-chip pump power (black dots), showing a threshold of 0.46 mW and a linear growth slope of 1.7% above the threshold for SARS by linearly fitting (red line).

When the pump wavelength was set as 1559.4 nm with above-threshold power, a Stokes Raman spectral line emerged at 1623.4 nm wavelength in the OSA, as depicted in Figure 4a. This spectral line is the first-order Stokes SRS emission, corresponding to the strong Raman response of the vibration frequency around 264 cm^−1^ [30]. As the pump power was gradually increased, the peak power of this spectral line grew by degrees, which is recorded in Figure 4b. It is clearly shown that the lasing action occurred at a pump power of ≥0.33 mW, which decreased by 47% from the record previously reported in TFLN microcavities [4,37,38,39]. And above the threshold, the dependence of the SRS peak power on the pump power revealed a linear growth with an efficiency as high as 40.6%, which increased by 24% from the corresponding records [39]. As the SRS process is a non-parametric optical process only relying on the requirement of a double-resonance condition that and free of the requirement of phase match, the SRS process can be observed in a broad bandwidth in principle. Thus, to confirm this nature of SRS, the pump wavelength was tuned to other WGMs within the same mode family to trigger SRS. When the pump wavelength was tuned from 1559.4 to 1556.2 nm (i.e., with a wavelength interval of one free spectral range), the corresponding Stokes SRS emission line shifted from 1623.4 to 1619.9 nm. When the pump wavelength was moved to ~1553.0 nm, the Stokes SRS emission line was observed at ~1616.5 nm. The threshold existences, linear growth above the threshold, and the broadband tunability are in agreement with the key features of microresonator-based Raman lasers [9,10]. 

This progress in SRS generation efficiency and the threshold enables SARS to be easily triggered at a low pump level. As seen in Figure 4c, both the first-order SRS and the first-order SARS signals appeared in the OSA when the on-chip pump power was increased to higher than 0.5 mW. The wavelength of the SARS spectral line was ~1500.3 nm. And the output power of the SARS signal also depends linearly on the on-chip pump power above the threshold, as shown in Figure 4d. The threshold for SARS was measured as 0.46 mW, and the linear growth efficiency for SARS was 1.7% above the threshold. This linear growth agrees well with theoretical prediction of SARS in the following explanation. In theoretical and experimental analysis [9,10], the intensity of the SRS signal linearly relies on the on-chip pump power, and the intensity of the SARS signal is also linearly dependent on the intensity of SRS. Therefore, the dependence of the power of the SARS signal on the on-chip pump power is linear. It is worth mentioning that the threshold for SARS is higher than that for SRS. This is due to the fact that the SARS generation efficiency is much lower than that of SRS, and the presence of SRS lasing is one of the prerequisites for the generation of SARS lasing. Thus, all these features agree well with the nature of SARS in microresonators [9]. 

**Figure 5:**
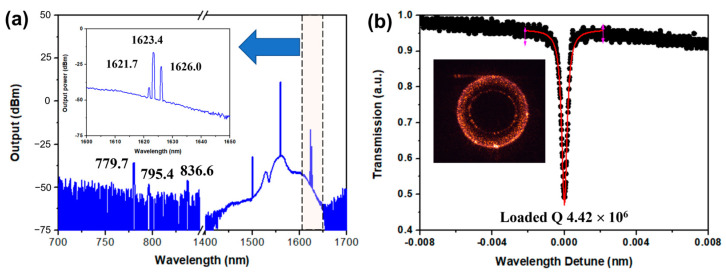
(**a**) The spectrum at a high pump power of 2 mW. Inset: the enlarged view image of the spectrum around the SRS signal at 1623.4 nm wavelength, showing two more peaks. (**b**) Transmission spectrum around the pump mode, showing a loaded Q factor of 4.42 × 10^6^ and a coupling efficiency of ~50%. Inset: The near infrared emission from the microdisk captured by the CCD camera with a working spectral window ranging from 400 to 1000 nm.

When we carefully examined the Raman vibration frequencies, there was a high Raman-gain broad envelope around 264 cm^−1^, with two neighboring peaks located at 255 and 276 cm^−1^ [40]. Once the on-chip pump power is higher than the thresholds for triggering these Raman frequencies within the broad Raman response bandwidth, more SRS Raman spectral lines can be excited. For example, when the on-chip pump power was raised to 2 mW, two more Raman spectral lines were observed at wavelengths of 1621.7 and 1626.0 nm, as shown in the inset of Figure 5a. The spectral interval of these two lines was narrower than one free spectral range of the fundamental WGMs of ~3.49 nm. As a result, these Raman lines should correspond to different spatial WGM families of the microdisk with high loaded Q factors. 

Additionally, some spectral signals also appeared around the 800 nm wavelength at this high pump level. Near infrared light emission from the microdisk was captured by the CCD camera with a working spectral width ranging from 400 to 1000 nm, showing a bright scattering signal, as depicted by the optical microscope image in the inset of Figure 5b. The spectrum collected from the output port of the fiber splitter gives clear evidence of the generation of a second harmonic signal at the ~779.7 nm wavelength, as shown in Figure 5a. The output power of the second harmonic signal at 779.7 nm wavelength was measured to ~3 μW. Moreover, a weak Raman line was detected at a wavelength of 795.4 nm with a total power of ~20 nW, which was generated based on a two-photon SRS process via the sum frequency generation of the pump light and the SRS lasing signal at 1621.4 nm [39,41,42], with a relationship of that 1/795.4 is equal to the sum of 1/1559.4 and 1/1623.4. The weak intensity of a second harmonic was insufficient to directly trigger SRS in the second harmonic wave band. This is due to the fact that the Q factor of the second harmonic mode is often much smaller than that of the pump mode because the shorter wavelength light often suffers from larger scattering loss [23,39]. As a result, to directly trigger the SRS process in the second harmonic waveband, a much higher power was required than in the 1560 nm waveband. Thus, the generated second harmonic signal was too weak to access the lasing threshold. Besides this Raman line, the other Raman line was also detected at a 836.6 nm wavelength with a total power of 30 nW, and this comb line should also be attributed to the other two-photon SRS process involved with an SRS lasing line at a 1804.9 nm wavelength. However, the wavelength of 1804.9 nm exceeded the OSA detection bandwidth ranging from 600 to 1700 nm. Therefore, this SRS emission line was not detected using this OSA.

## 5. The Discussion

The generation of the highly efficient SRS laser and the low-threshold SARS laser in the TFLN microdisk should be attributed to the cavity-enhanced excitation of the strong Raman-gain vibration frequency around 264 cm^−1^. Thus, it is necessary to measure the loaded Q factors of the WGMs. The pump mode was characterized with a Lorentz shape, as shown in Figure 5b. The loaded Q factor (*Q*_L_) of the pump mode was determined as 4.42 × 10^6^. And the over-coupled efficiency was measured to be ~50%. Thus, the intrinsic Q factor was calculated as 3.0 × 10^7^. This ultra-high Q factor will result in a significant boost to the built-up circulating light intensity of the pump light, the SRS signal, and the SARS signal within the small microdisk volume, leading to a high SRS efficiency under the excitation of the strong Raman-gain vibration frequency of TFLN. In principle, the threshold of SRS is inversely proportional to the factor of $Q_{L}^{2}/V$ [10], where *V* is the mode volume with value $V\leq 2\pi Rn\cdot \lambda^{2}$. Here, the parameters of *R*, *n*, and *λ* are the radius of the microdisk, the effective refractive index ~2.0, and the pump wavelength, respectively. It is worth noting that the fabricated microdisk resonator simultaneously possesses ultra-high Q factors and a small mode volume, which gives rise to a dramatic reduction in the pump threshold for the SRS lasing, which in turn triggers the generation of the low-threshold SARS laser on-chip. 

To further increase the SARS efficiency, the dispersion of the microdisk should be controlled more carefully to align the stronger Raman photon involved with a vibration frequency of 255 cm^−1^ to the high-Q WGMs for achieving higher Raman gain. It is also worth noting that we can improve the loaded Q factors of the microdisk to further increase the SARS efficiency either by optimizing the coupling condition to critical coupling or by replacing the TFLN wafer produced by ion-slicing with the one produced by chemo-mechanical polishing for thinning [43]. This is due to the fact that the latter wafer is free of ion implantation, which would induce lattice damage and in turn make the TFLN wafer suffer from higher inner scattering loss. If such a lattice-damage-free TFLN wafer is employed to fabricate a high-Q microdisk by the femtosecond laser PLACE technique, an ultrasmooth surface with an average surface roughness of 0.1 nm can be achieved [43]. Moreover, besides the suppression of the surface scattering loss, unavoidable inner lattice damage produced by the most common nanofabrication method using argon ion milling is avoided by employing the PLACE technique. As a result, intrinsic Q factors have been reported as high as 1.23 × 10^8^, and the loaded Q factors can reach 0.75 × 10^8^ [43]. Therefore, if such high-Q TFLN microresonators are dispersion engineered to excite the strong Raman vibration frequencies, a significant reduction in the threshold for SARS and a high increase in the SARS efficiency can be achieved. Furthermore, to achieve a monolithically integrated Raman laser, the suspended TFLN microdisk should be replaced with an unsuspended microdisk sitting on the silicon dioxide layer to achieve better mechanical stability and portability, and the tapered fiber should be replaced with a side-coupled TFLN bus waveguide with suitable coupled spacing to achieve critical coupling. 

## 6. Conclusions

To summarize, a low-threshold anti-Stokes laser was demonstrated in a high-Q TFLN microdisk at room temperature for the first time, to the best of our knowledge. The Stokes laser and the anti-Stokes laser operated with thresholds of only 0.33 and 0.46 mW, respectively. This on-chip upconverted anti-Stokes Raman laser was attributed to the excitation of strong Raman-gain vibration frequency by aligning the Raman photon to the ultra-high-Q (>4 × 10^6^) WGMs in the TFLN microdisk fabricated by the femtosecond laser PLACE technique. It is worth noting that the pure TFLN cannot provide optical gain for gain directly, because it is an indirect band-gap material [20]. When combining this blue-shifted laser with the strong electro-optic, piezoelectric, acousto-optic, and second-order nonlinear properties of the TFLN, there will be great improvement in the performance and functionality in TFLN photonic integration, paving the way for high-speed optical information processing and quantum information processing.

## Data Availability

Data are contained within the article.
